# Complete Genome Sequence of Megamonas funiformis JCM 14723^T^

**DOI:** 10.1128/MRA.00142-20

**Published:** 2020-04-16

**Authors:** Dieter M. Tourlousse, Mitsuo Sakamoto, Takamasa Miura, Koji Narita, Akiko Ohashi, Yoshihito Uchino, Atsushi Yamazoe, Keishi Kameyama, Jun Terauchi, Moriya Ohkuma, Hiroko Kawasaki, Yuji Sekiguchi

**Affiliations:** a Biomedical Research Institute, National Institute of Advanced Industrial Science and Technology (AIST), Tsukuba, Ibaraki, Japan; b Microbe Division/Japan Collection of Microorganisms, RIKEN BioResource Research Center, Tsukuba, Ibaraki, Japan; c Biological Resource Center, National Institute of Technology and Evaluation (NITE), Kisarazu, Chiba, Japan; d Japan Microbiome Consortium (JMBC), Osaka, Osaka, Japan; Loyola University Chicago

## Abstract

We announce the complete genome sequence of Megamonas funiformis JCM 14723^T^ (YIT 11815^T^). The genome consists of a circular chromosome (2,522,577 bp, 31.5% G+C content) and a plasmid of 46,189 bp (29.4% G+C content). The genome was predicted to contain 6 rRNA operons, 53 tRNA genes, and 2,440 protein-coding sequences.

## ANNOUNCEMENT

Megamonas funiformis, originally isolated from human feces of healthy Japanese males (1), is one of three described species of the *Megamonas* genus within the family *Selenomonadaceae*. Limited information is available about the prevalence and role of this species in the human gastrointestinal tract. One study reported reduced abundance in the feces of patients with multiple system atrophy (2). To address the lack of complete genome sequences of *M. funiformis*, we determined a complete genome sequence of *M. funiformis* JCM 14723^T^ (YIT 11815^T^), the authentic type strain of this species (1).

Cells of strain JCM 14723^T^ were obtained from the Japan Collection of Microorganisms and cultured at 37°C for 36 h in modified Gifu anaerobic medium (GAM broth) with 1% glucose under an N_2_ atmosphere. The EZ1 DNA tissue kit (Qiagen) was used for DNA purification, following cell lysis by bead beating. The TruSeq Nano DNA kit was used to generate short-read libraries, and sequencing was performed on a MiSeq instrument using v2 chemistry (2 × 251-bp reads); estimated coverage was ∼320×. Quality control of the reads was performed with Trimmomatic v0.38 (3). Libraries for Oxford Nanopore Technologies (ONT) sequencing were prepared with a ligation sequencing kit (SQK-LSK109) and native barcoding expansion pack (EXP-NBD104). Sequencing employed an R9.4.1 flow cell (FLO-MIN106) and the MinION device. Base calling of ONT reads was performed with Guppy v3.1.5 (ONT) in high-accuracy mode, with simultaneous library demultiplexing and read trimming; reads with a Q score of <9 and size of <1,000 bp were discarded using NanoFilt v2.5.0 (4). Filtlong v0.2.0 (https://github.com/rrwick/Filtlong) was then used to identify high-quality ONT reads by using the Illumina reads as references and discarding the 10% poorest read bases. A total of 88,322 ONT reads (*N*_50_, 5,833 bp; coverage, ∼170×) were used to generate a long-read assembly using Flye v2.5 (5). This assembly was used together with the Illumina reads (3,364,679 total reads) to generate a hybrid assembly using Unicycler v0.4.7 (6), run with default settings, including error correction using Pilon. Annotation was performed with the NCBI Prokaryotic Genome Annotation Pipeline v4.11 (7).

The genome of *M. funiformis* JCM 14723^T^ consists of a 2,522,577-bp chromosome (G+C content, 31.5%) and an extrachromosomal element of 46,189 bp (G+C content, 29.4%); both elements were indicated as circular by Flye/Unicycler. The 46-kbp element may represent a conjugative plasmid based on the identification of genes encoding relaxase, the type IV secretion system, and the type IV coupling protein by oriTfinder (8). The chromosome was predicted to contain 6 sets of rRNA genes and 53 tRNA genes and encode 2,385 proteins; the plasmid harbored 55 protein-coding sequences. This genome sequence will contribute to our understanding of the ecology of *M. funiformis* and its interactions with the human host by providing a picture of the functional potential encoded in the genome of *M. funiformis* JCM 14723^T^.

### Data availability.

This genome sequence is available in DDBJ/EMBL/GenBank under accession numbers CP048627 (chromosome) and CP048628 (plasmid). Raw ONT and Illumina sequencing reads have been deposited in the Sequence Read Archive (SRA) under accession numbers SRR10968457 and SRR10968460, respectively.
